# Three non-autonomous signals collaborate for nuclear targeting of CrMYC2, a *Catharanthus roseus *bHLH transcription factor

**DOI:** 10.1186/1756-0500-3-301

**Published:** 2010-11-12

**Authors:** Sabah Hedhili, Marie-Véronique De Mattei, Yoan Coudert, Isabelle Bourrié, Yves Bigot, Pascal Gantet

**Affiliations:** 1 Université François Rabelais, UFR des Sciences et Techniques, Unité sous Contrat reconnue par l'Institut National de la Recherche Agronomique, Facteurs de Transcription et Ingénierie Métabolique Végétale, Biomolécules et Biotechnologies Végétales, EA 2106, Parc de Grandmont, 37200 Tours, France; 2 Université François Rabelais, UFR des Sciences et Techniques, Génétique, Immunothérapie Chimie et Cancer, UMR CNRS 6239, Parc de Grandmont, 37200 Tours, France; 3 Université Montpellier 2, UMR 1098, Développement et Amélioration des Plantes, Place Eugène Bataillon, CC002, 34095 Montpellier Cedex 5, France

## Abstract

**Background:**

CrMYC2 is an early jasmonate-responsive bHLH transcription factor involved in the regulation of the expression of the genes of the terpenic indole alkaloid biosynthesis pathway in *Catharanthus roseus*. In this paper, we identified the amino acid domains necessary for the nuclear targeting of CrMYC2.

**Findings:**

We examined the intracellular localization of whole CrMYC2 and of various deletion mutants, all fused with GFP, using a transient expression assay in onion epidermal cells. Sequence analysis of this protein revealed the presence of four putative basic nuclear localization signals (NLS). Assays showed that none of the predicted NLS is active alone. Further functional dissection of CrMYC2 showed that the nuclear targeting of this transcription factor involves the cooperation of three domains located in the C-terminal region of the protein. The first two domains are located at amino acid residues 454-510 and 510-562 and contain basic classical monopartite NLSs; these regions are referred to as NLS3 (KRPRKR) and NLS4 (EAERQRREK), respectively. The third domain, between residues 617 and 652, is rich in basic amino acids that are well conserved in other phylogenetically related bHLH transcription factors. Our data revealed that these three domains are inactive when isolated but act cooperatively to target CrMYC2 to the nucleus.

**Conclusions:**

This study identified three amino acid domains that act in cooperation to target the CrMYC2 transcription factor to the nucleus. Further fine structure/function analysis of these amino acid domains will allow the identification of new NLS domains and will allow the investigation of the related molecular mechanisms involved in the nuclear targeting of the CrMYC2 bHLH transcription factor.

## Background

*Catharanthus roseus *L. G. Don. produces terpenoid indole alkaloids (TIAs). Some TIAs have pharmaceutical properties, such as the hypotensive compound ajmalicine and the anticancer agents vinblastine and vincristine. The biosynthesis of TIAs is induced by jasmonate [1], and the expression of several genes encoding enzymes of the TIA biosynthesis pathway are coordinately regulated by the APETALA2 (AP2)-domain ORCA3 (octadecanoid derivative-responsive *Catharanthus *AP2-domain) transcription factor (TF) in response to this hormone [2]. The *Orca3 *gene is itself regulated by jasmonate and possesses a jasmonate-responsive element (JRE) in its promoter [3]. This JRE is composed of 1) a qualitative region that switches on *Orca3 *expression in response to jasmonate and 2) a quantitative element that acts as an enhancer of transcription. The qualitative region is a G-box-like element (AACGTG). An early jasmonate-responsive bHLH TF, CrMYC2 (AF283507), binds this qualitative element and activates the expression of the *Orca3 *gene (our unpublished data). CrMYC2 was isolated by a yeast one-hybrid screen using a G-box sequence (CACGTG) as bait [4]. Transcription factors such as CrMYC2 can be used as metabolic engineering tools to improve the production of valuable secondary metabolites in plant cells [5]. However, the use of transcription factors as metabolic engineering tools supposes that the transcription factor is properly targeted to nucleus when overexpressed in the cell.

CrMYC2 belongs to the basic helix-loop-helix (bHLH) TF family. The bHLH TF family comprises 162 genes in *Arabidopsis thaliana *and 167 genes in *Oryza sativa*, but the functions of only few of these genes have been characterized [6-8]. In particular, protein regions involved in the targeting of bHLH transcription factors to the nucleus have been functionally identified in only a few of them [7-18]. The nuclear import of proteins is often mediated by specific sequences, called nuclear localization signals (NLSs). NLSs are recognized by receptors (importin α and importin β) that allow the interaction of the protein with the nuclear pore and the translocation of the protein complex into the nucleus [19]. Several NLS sequences have been characterized. Short stretches of basic amino acids (aa), including both monopartite ((K/R) _4/6_) and bipartite ((K/R) _2 _X_10_-_12 _(K/R) _3_) sequences, are present in various nuclear proteins [20-23]. In addition, structure/function analysis of different nuclear proteins has revealed that a variety of non-conserved sequences are also involved in nuclear targeting [24-26].

Concerning plant bHLH TFs, only one structure/function analysis of nuclear localization, the one completed for the maize R protein, has been performed in detail. The maize R protein is involved in the control of anthocyanin biosynthesis in various organs in maize [27]. The authors demonstrated that this protein contains three different NLSs (A, M, and C). NLS-A contains arginine residues but not lysine (characteristic of some viral NLSs). NLS-C contains hydrophobic amino acids surrounding basic residues, and NLS-M is rich in basic amino acids, similar to classical monopartite NLSs. The truncated N-terminal or C-terminal part of this protein can be targeted to nucleus only if both NLS-A and NLS-M or NLS-C and NLS-M are simultaneously present [9].

In this paper, we present a structure/function analysis of the nuclear targeting of CrMYC2. Different sequences from CrMYC2 were fused with GFP and transiently expressed in epidermal onion cells. Our data show that none of the basic aa-rich sequences identified as putative NLSs are functional alone and that the nuclear targeting of CrMYC2 involves cooperation among three different domains of the C-terminal part of the protein.

## Methods

### Plasmid construction

The cassette containing the CaMV 35S promoter, a multicloning site and the *mgfp5 *sequence followed by a *T-Nos *terminator sequence was removed from pCambia-1302 with SphI and inserted into the SphI site of pEMBL18. This plasmid construct was called GFP-pEMBL18. The GFP-pEMBL18 plasmid was used to clone, in phase with the 3' extremity of the *mgfp5 *coding sequence, the full-length *Crmyc2 *cDNA (GenBank accession number AF283507), *Crmyc2 *deletion mutants, or annealed oligonucleotides corresponding to putative NLSs. These putative NLSs were identified by bioinformatics analysis (http://wolfpsort.org/) or a manual search for stretches of basic amino acids similar to classical mono- and bipartite- NLSs [26,28-30].

**Figure 1:**
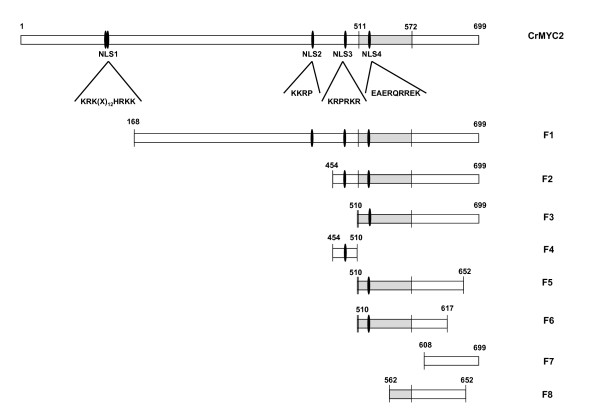
**Schematic representation of the full-length CrMYC2 protein showing the positions of the potential nuclear localization signal sequences (NLS 1, 2, 3, and 4) and of the regions of CrMYC2 used to make the GFP fusions**. NLS: nuclear localization signal, CrMYC2: full-length CrMYC2 sequence, F1 to F8: partial CrMYC2 sequences used for fusion to GFP. Grey box: bHLH domain. Single oval shape: monopartite NLS. Double oval shape: bipartite NLS.

The complete *Crmyc2 *cDNA or deletion derivatives were amplified by PCR using specific primers containing at their 5' end a NcoI site for the forward primer and an SpeI site for the reverse primer except for the full-length *Crmyc2 *and the deletion mutant *F1 *in which a NcoI restriction site was added to both the forward and reverse primers because an SpeI site is present in the *Crmyc2 *sequence. PCR cycling conditions were as follows: 94°C for 4 min (1 cycle) followed by 94°C for 1 min, an annealing step at various temperatures depending on the *T*_m _of the primers used for 1.5 min, and 72°C for 1 min (30 cycles), with a final extension step at 72°C for 5 min. PCRs were performed in a final volume of 25 μl with 0.25 U of *Taq *polymerase and 1 × MgCl_2_-free buffer (Promega), 2 mM MgCl_2_, 200 nM of each dNTP, appropriate oligonucleotides (1 μM each) and 5 ng of *Crmyc2 *cDNA in pGEM-T Easy (Promega). PCR products were cloned into pGEM-T Easy (Promega), removed by NcoI/SpeI (or with only NcoI for full-length *Crmyc2 *cDNA and deletion mutant *F1*) and subcloned into the GFP-pEMBL18 plasmid cut with NcoI and SpeI. Double-stranded sequences encoding putative NLSs of CrMYC2 with cohesive 5'-NcoI and 3'-SpeI ends were reconstituted from single strand oligonucleotides (Oligo Express, France) and cloned into the GFP-pEMBL18 plasmid digested with NcoI and SpeI. Correct orientation and fusion of the cloned sequences and the *gfp *sequence were verified by sequencing (MWG Biotech, Germany). The sequences of the primers used are given in Table 1. The different protein fragments or NLS sequences fused with GFP are schematically shown in Figure 1. In addition, an N-terminal fusion of CrMYC2 with GFP was obtained by cloning the full-length *Crmyc2 *cDNA into the pTH2^BN ^plasmid [31], by a similar procedure. *Crmyc2 *was inserted into the XhoI site of the pTH2^BN ^plasmid.

**Table 1:** Oligonucleotides used for the amplification of full-length or deletion mutants of *Crmyc2 *or for the reconstitution of sequences encoding the putative NLSs of the CrMYC2 protein

Fusion protein name	Nucleotide sequences of primers used to amplify the *Crmyc2 *fragments or to reconstitute the short coding sequences of the NLSs	
CrMYC2	forward	5'-CCGCTCGAGATGACGGACTAT-3'
	
	reverse	5'-CCGCTCGAGTCATACCAAGAG-3'

F1	forward	5'-CCGCCATGGGCACCGCCGATGATGCA-3'
	
	reverse	5'-CCGCCATGGGTACCAAGAGCCTCATCG-3'

F2	forward	5'-CCGCCATGGGGGTAGTTTTGCCCTCTACT-
	
	reverse	5'-CCGACTAGTTACCAAGAGCCTCATCGAGT-3'

F3	forward	5'-CCGCCATGGGAAGGGAAGAGCCATTGAAT-3'
	
	reverse	5'-CCGACTAGTTACCAAGAGCCTCATCGAGT-3'

F4	forward	5'-CCGCCATGGGGGTAGTTTTGCCCTCTACT-3'
	
	reverse	5'-CCGCCATGGCCATTTGCAGGCTTTCTCCC 3'

F5	forward	5'-CCGCCATGGGAAGGGAAGAGCCATTGAAT-3'
	
	reverse	5'-CCGACTAGTTAATTCAAGATCAAGAT-3'

F6	forward	5'-CCGCCATGGGAAGGGAAGAGCCATTGAAT-3'
	
	reverse	5'-CCGACTAGTATCAATATCCATATCCAAGTTACC-3'

F7	forward	5'-CCGCCATGGGTAACTTGGATATGGATATTG-3'
	
	reverse	5'-CCGACTAGTTACCAAGAGCCTCATCGAGT-3'

F8	forward	5'-CCGCCATGGCTAAACTTCAA-3'
	
	reverse	5'-CCGACTAGTTAATTCAAGATCAAGAT-3'

NLS1	forward	5'-CATGGGGAAGAGGAAGAATTCGTCTTCCGCGAGT TCTTTTGCAGAACAGGAACACAGAAAGAAAA-3'
	
	reverse	5'-CTAGTTTTCTTTCTGTGTTCCTGTTCTGCAAAAGAA CTCGCGGAAGACGAATTCTTCCTCTTCCC-3'

NLS2	forward	5'-CATGGAGAACAAGAACAAGAAAAGGCCATCTA-3'
	
	reverse	5'-CTAGTAGATGGCCTTTTCTTGTTCTTGTTCTC-3'

NLS3	forward	5'-CATGGAGAAACGGCCTAGGAAGAGGA-3'
	
	reverse	5'-CTAGTCCTCTTCCTAGGCCGTTTCTC-3'

NLS4	forward	5'-CATGGAGGCAGAGAGGCAGAGGAGGGAGAAGTTGA-3'
	
	reverse	5'-CTAGTCAACTTCTCCCTCCTCTGCCTCTCTGCCTC-3'

Underlined sequences represent the positions of the restriction sites used for cloning the PCR products into the GFP-pEMBL18 or pTH2BN vectors.

### Biolistic transformation of onion epidermal cells

Commercially available white onions were surface disinfected with 70% ethanol. After dissection, the internal epidermis layer was peeled and placed face up on solid MS medium (8 g agar/liter) without vitamins (Duchefa, M0222) in 55-mm diameter Petri dishes. The particle bombardment transformation was carried out using a PDS-1000 BioRad system with 1800 Psi rupture disks (BioRad) under reduced pressure of 30 mm Hg. Plasmid DNA (10 μg) was coated on tungsten M-25 particles (BioRad) in 25 μL of 2.5 M CaCl_2 _and 10 μL of 0.1 M spermidine. Particles were homogenized for 2 min by ultra-sonication and sedimented by gravity for 15 min. Supernatant (15 μL) was removed, and after a short sonication step, 4 μL of the remaining particle mixture was deposed on a macrocarrier disk (BioRad). During bombardments, samples were placed 6 cm from the stopping screen. Onion cells were transformed with the plasmids carrying different CrMYC2 (full length or deletion) GFP fusions and NLS-GFP fusions under the control of the CaMV 35S promoter. After transformation, the samples were incubated for 12 h in the dark at 25°C.

### Epifluorescence microscopy

Epidermal onion cells were observed with an epifluorescence microscope (Olympus BX51) equipped with a digital camera (Olympus DP50) and the corresponding software (OLYMPUS ANALYSIS). The GFP fluorescence was imaged with a blue excitation filter set (460-490 nm excitation filter, 515 nm cut-off filter). After staining with DAPI (600 nM), fluorescence was observed with UV filter set (360 nm excitation filter, 460 cut-off filter).

## Results

### Four putative NLSs are present in the CrMYC2 sequence

The deduced amino acid sequence from the open reading frame of the *Crmyc2 *cDNA is characterized by a conserved basic helix-loop-helix (bHLH) domain (residues 511-572) typical of the bHLH class of transcription factors. The basic domain of bHLH transcription factors is involved in the interaction with DNA [32-35], and the HLH motif is involved in protein dimerization [36,37] and participates in DNA binding specificity [38,39].

**Figure 2:**
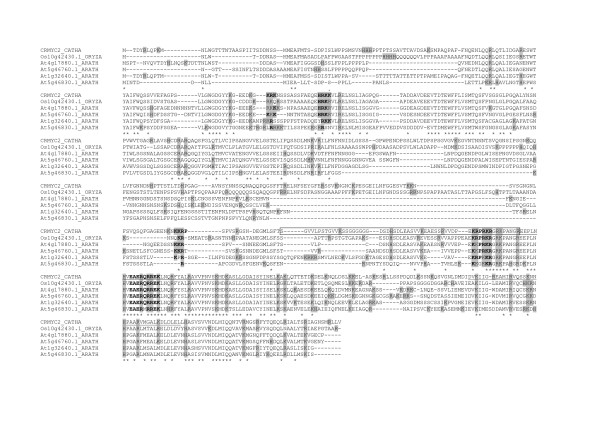
**Alignment of amino acid sequences of CrMYC2, AtMYC2/RAP1/AtbHLH006/At1g32640.1, At4g17880.1, At5g46760.1, At5g46830.1 and Os10g42430.1**. Bold letters correspond to conserved amino acids residues within a putative NLS. Basic amino acids are boxed in grey. Asterisks under sequences correspond to positions where the amino acid is the same for all sequences. Boxed or underlined sequences indicate the three regions involved in CrMYC2 nuclear targeting.

The search for NLSs in the CrMYC2 sequence using protein subcellular localization prediction software (WoLF PSORT http://wolfpsort.org/) revealed the presence of a unique putative monopartite NLS domain (KRPRKR, amino acids 498-503) in the C-terminal part of the protein, corresponding to a short sequence just before the bHLH domain (Figure 1). This putative NLS sequence was named NLS3. Further searches for domains rich in basic amino acids located three other domains that are similar to NLSs previously characterized in other proteins [26,40] (Figures 1 and 2). NLS1, KRK(X)_12_HRKK, which is located between residues 137 and 155, is bipartite; NLS2, KKRP, is located between amino acids 434 and 437; and NLS4, EAERQRREK, is located between amino acids 519 and 527 within the bHLH domain. Both NLS1 and NLS4 are monopartite.

In an attempt to verify whether these NLS candidates were conserved in related species, we determined if these NLSs are present in the sequences of close orthologs in the *A. thaliana *and *O. sativa *genomes using the GreenPhyl database (http://greenphyl.cirad.fr/v2/cgi-bin/index.cgi). The alignment of the CrMYC2 sequence with *A. thaliana *AtMYC2/RAP1/AtbHLH006/At1g32640.1, rice 0s10g42430.1 orthologs and three other highly phylogenetically related *A. thaliana *sequences revealed that NLS1 and NLS2 were weakly conserved. In contrast NLS3 and NLS4 were well conserved (Figure 2), thus suggesting that they were conserved during evolution based on their function.

### Putative NLSs identified in the CrMYC2 protein are not functional alone in plant cells

**Figure 3:**
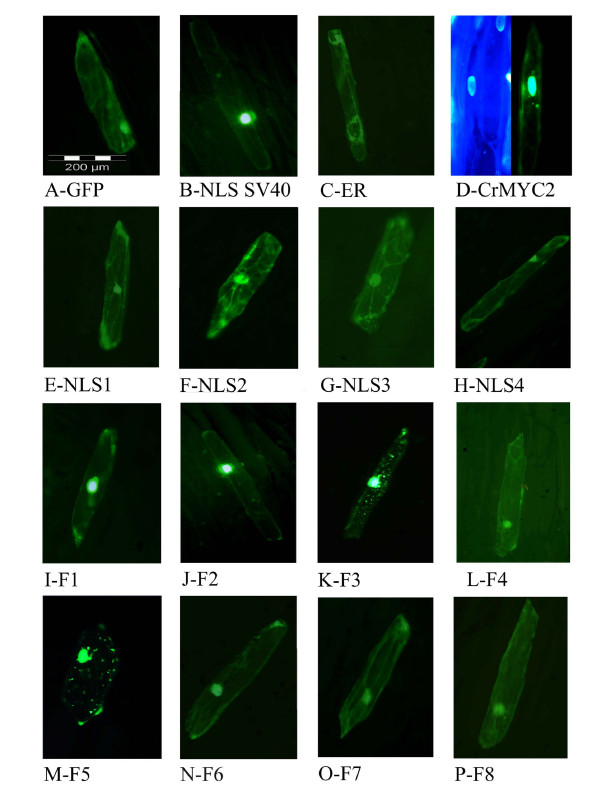
**Subcellular localization in epidermal onion cells of fusions of GFP with full-length CrMYC2, with different putative nuclear localization signals and with deletion mutants of CrMYC2**. The green fluorescence of the GFP protein was visualized by epifluorescence microscopy. A: control with GFP alone. B: control with GFP fused to the NLS of the SV40 large T antigen. C: control with GFP fused to an endoplasmic reticulum retention signal. D: right, GFP fused to full-length CrMYC2, left: corresponding DAPI staining,. E-H: GFP fused with different putative NLS sequences from CrMYC2, 1, 2, 3 and 4, respectively; I-P: GFP fused with different deletion mutants of CrMYC2 (F1 to F8).

To verify whether these motifs were functional NLSs, they were fused to the N-terminal extremity of GFP and transiently expressed in onion epidermal cells. In addition, two controls were assayed: a GFP fusion with the short monopartite NLS "PKKKRKV" from the SV40 large T antigen, which is ubiquitously functional among eukaryotes, and a GFP fused with a "KDEL" motif that is an endoplasmic reticulum retention signal. The data presented in Figure 3 depict the localization of each GFP fusion in onion cells. In controls, the GFP alone was homogeneously distributed in the cytoplasm and the nucleus (Figure 3A) due to passive diffusion through the nuclear pores, whereas GFP fused to the NLS of SV40 large T antigen was actively concentrated in the nucleus (Figure 3B), and GFP fused to the endoplasmic reticulum retention signal was only located in the cytoplasm (Figure 3C). Figures 3E to 3H show that the cellular localization of each of the four fusions made with the putative CrMYC2 NLS was similar to the cellular localization observed for GFP alone, both in the nucleus and the cytoplasm. This finding suggests that none of these four putative NLS sequences is individually able to drive active nuclear translocation of the GFP fusions in plant cells.

### The N-terminal part of *CrMYC*2 is not required for its nuclear localization

To identify the region of CrMYC2 involved in its translocation to the nucleus, full-length and deletion mutants of CrMYC2 were fused to GFP (Figure 1) and expressed in onion cells. The results are presented in Figure 3. All of the data were obtained with fusions made at the N-terminal extremity of GFP except for the full-length CrMYC2 protein, which was successfully expressed only when it was fused to the C-terminal end of GFP. The fusion between GFP and full-length CrMYC2 exhibited a different localization compared to the GFP alone: the fluorescence was mostly observed in the nucleus but also, some small spots in the cytoplasm were observed (Figure 3D right). The nucleus localization of CrMYC2 was confirmed by staining the genomic DNA in cells with DAPI (Figure 3D left). Similar data were obtained with transformation experiments using a lower amount of the expression plasmid (2 μg or 4 μg), suggesting that the cytoplasmic spots were not the result of the overexpression of the GFP-CrMYC2 fusion protein. The F1 and F2 fusions accumulated strongly in the nucleus (Figure 3I-J). These data show that the N-terminal part of the protein between residues 1 and 454 was not strictly required for the nuclear import of CrMYC2 into the nucleus. In addition, the amino acids sequence 1-168 could bear a signal responsible for the retention of the GFP fusions in spots in the cytoplasm.

### Different domains act in collaboration for *CrMYC2 *subcellular localization

To localize the other domains involved in CrMYC2 nuclear targeting, further functional dissection of the C-terminal part (aa 454-699) was done.

The F3 fusion had a cellular localization pattern similar to that of the GFP-CrMYC2 fusion (Figure 3K). This suggested that the 454-510 region of the protein contains a signal allowing the complete targeting of the protein to the nucleus and/or preventing its accumulation in spots in the cytoplasm. Nevertheless, when expressed alone in fusion with GFP (F4), this 454-510 sequence did not have an effect on the subcellular localization of the GFP fluorescence compared to the expression of GFP alone (Figure 3L and 3A). This last result indicated that the signal contained in this part of the protein collaborates with other signals located in the 510-699 region of CrMYC2 for its nuclear targeting.

The subcellular localization of the F5 fusion, corresponding to the 510-699 region lacking the 47 C-terminal amino acids, was similar to the cellular localization of the F3 fusion, showing that the C-terminal 47 amino acids of CrMYC2 did not play any role in subcellular localization (Figure 3M). When an additional 35 C-terminal amino acids (F6 fusion) were removed (Figure 3N), the localization of the green fluorescence in the cell was identical to the one observed for GFP alone, suggesting that the 617-652 aa portion of the protein participates in the nuclear targeting of CrMYC2. Nevertheless, the F7 fusion (608-699), which includes this region but lacks the main N-terminal part of the protein, failed to be actively targeted to the nucleus (Figure 3O). When the 510-562 portion was removed (F8 fusion) from F5, the subcellular localization was identical to the one of GFP alone (Figure 3P). This result suggested that another domain located in the 510-652 part of the protein was also necessary for proper nuclear targeting. All of these data showed that the targeting of the CrMYC2 protein to the nucleus involved different domains located at aa 454-562 and aa 617-652 and that these domains acted in collaboration.

## Discussion and conclusions

In this study, we investigated the nuclear targeting of CrMYC2, a *C. roseus *bHLH TF. The contribution of the different domains of this protein in nuclear targeting was evaluated in epidermal onion cells transiently expressing GFP protein fusions.

Proteins with a molecular weight less than 40-60 kDa are able to enter the nucleus by passive diffusion [40]. This was illustrated by the cellular localization of GFP (MW: 28 kDa) alone, which was both cytoplasmic and nuclear. This default distribution allows the detection of NLSs and of motifs that sequester the protein in the cytoplasm. This notion was validated by the results obtained for GFP fused with the NLS from the SV40 large T antigen, which was primarily localized in the nucleus, and for the fusion of GFP with a "KDEL" endoplasmic reticulum retention signal, which was localized only in the cytoplasm (Figure 3B-C).

The search for conserved basic NLS motifs in the CrMYC2 sequence revealed four putative NLSs [20,30,41]: one bipartite, NLS1, and three monopartite NLSs: NLS2, NLS3 and NLS4. None of these four NLSs were able to actively target GFP to the nucleus individually. When the CrMYC2 sequence was compared to the sequence of two orthologs, *A. thaliana *AtMYC2/RAP1/AtbHLH006/At1g32640.1 and *O. sativa *Os10g42430.1, and with three other phylogenetically related *A. thaliana *bHLH proteins (Figure 2), we found that the NLS1 and NLS2 sequences are weakly conserved. On the contrary, the NLS3 and NLS4 sequences are well conserved among the six proteins. In addition, NSL1 and NLS2 are located in the 1-454 N-terminal part of CrMYC2, which was not necessary for the nuclear targeting of CrMYC2, whereas NLS3 and NLS4 are located in the C-terminal part of the protein, which contained the signals necessary for nuclear targeting. In conclusion, NLS1 and NLS2 are likely not involved in the nuclear targeting of CrMYC2, whereas NLS3 and NLS4 could play a role in the nuclear targeting of CrMYC2 by acting in a collaborative way.

The functional dissection of CrMYC2 revealed that the C-terminal region (aa 454-699) of the protein contains the signals that are necessary and sufficient for nuclear targeting of the protein. This observation is in accordance with previous data obtained for the orthologous *A. thaliana *protein AtMYC2. Using GFP-protein fusions expressed in tobacco and *A. thaliana *cells, it has been shown that the full-length AtMYC2-GFP fusion is actively targeted to the nucleus, whereas a mutant lacking the C-terminal part comprising the bHLH domain has the same subcellular distribution profile as GFP alone [8]. These results suggest that conserved motifs located in the C-terminal region of the protein are involved in the nuclear targeting of the CrMYC2 and AtMYC2 proteins.

To identify the motifs involved in nuclear targeting, further functional analysis of the C-terminal part of CrMYC2 was done. The data obtained showed that three regions, aa 454-510, aa 510-562 and aa 617-652, are necessary for the nuclear targeting of CrMYC2, but individually, none of these sequences was able to target the GFP fusion to the nucleus, suggesting that these regions cooperate to drive CrMYC2 to the nucleus. The bHLH TF family is widespread in eukaryotic organisms and is a large gene family in *A. thaliana *and *O. sativa *[42-45]. Nevertheless, few studies have been devoted to the identification of the NLS(s) involved in their nuclear targeting. One of the more detailed studies concerns the bHLH TF encoded by the *R *gene from maize [9]. Three active NLS have been identified in this protein: NLS-A (aa 100-109, GDRRAAPARP), NLS-M (aa 419-428, MSERKRREKL) and NLS-C (aa 598-610, MISESLRKAIGRK). NLS-M and NLS-C are independently sufficient to direct the GUS protein to the nucleus. Interestingly, further analysis demonstrated that these NLSs act cooperatively to target the R protein to the nucleus. Efficient targeting is obtained only if both NLS-A and NLS-M or NLS-C and NLS-M are present. NLS-M corresponds to the basic part of the bHLH and is similar to NLS4 in CrMYC2. Some authors have suggested that the DNA-binding function of several classes of transcription factors have evolved to have NLS functions [29,46]. The two other NLSs, NLS-A and NLS-C, are not found in CrMYC2. Although the NLSs of these two plant bHLH TF are different, in both cases the cooperation of different regions of the protein is necessary to target the protein to the nucleus. In CrMYC2, the regions of aa 454-510 and aa 510-562 contain NLS3 and NLS4, respectively. These two NLS sequences are well conserved among five related TF from *A. thaliana *and *O. sativa *and are likely the functional NLS sequences of these regions. The aa 617-652 region does not contain sequences reminiscent of known NLSs. Nevertheless, this fragment is located in a very well-conserved sequence located after the bHLH domain between aa 614 and 690 (Figure 2). The aa 617-652 region is rich in conserved basic amino acids and could correspond to a new non-autonomous NLS. A variety of non-conventional signals, very diverse in sequences, have been described for the nuclear targeting of several proteins [24,47,48] including bHLH related transcription factors [49]. Full-length CrMYC2, F3 and F5 GFP fusions were localized in the nucleus but were also found in concentrated spots into the cytoplasm. We do not have adequate evidence to know if this profile corresponds to the artificial retention of these fusion proteins in vesicles or organelles or if this localization pattern has biological significance. A recent study reported the localization of a tobacco bHLH protein, NtWIN4, in plastids, suggesting the possibility that this nuclear transcription factor evolved from a plastid-resident regulatory factor [48]. However, the sequence responsible for NtWIN4 plastid targeting is not present in CrMYC2.

Further studies using point mutation experiments will be necessary to determine the amino acids important for the NLS function of the three functional conserved NLS domains identified in the CrMYC2 transcription factor.

## List of abbreviations

aa: amino acid; bHLH: basic helix-loop-helix; DAPI: 4'-6-Diamidino-2-phenylindole; GFP: green fluorescent protein; NLS: nuclear localization signal; ORCA3: Octadecanoid derivative-responsive *Catharanthus *AP-2 domain 3; TF: transcription factor; TIA: terpenoid indole alkaloids.

## Competing interests

The authors declare that they have no competing interests.

## Authors' contributions

SH, MVDM, YC, IB, and PG contributed to the realization of experiments; SH, YB, and PG conceived the study and participated in its design and coordination and helped to draft the manuscript. All authors read and approved the final manuscript.
